# Digital Tools for Seizure Monitoring and Self-Management in Epilepsy: A Narrative Review

**DOI:** 10.3390/jcm14248701

**Published:** 2025-12-09

**Authors:** Ekaterina Andreevna Narodova

**Affiliations:** Department of Neurology, Prof. V.F. Voyno-Yasenetsky Krasnoyarsk State Medical University, Krasnoyarsk 660022, Russia; katya_n2001@mail.ru; Tel.: +7-902-924-89-95

**Keywords:** epilepsy, drug-resistant epilepsy, seizure monitoring, digital health, mobile health application, medication adherence, patient self-management, wearable devices, seizure detection

## Abstract

**Background/Objectives:** Medication non-adherence and incomplete seizure documentation remain major challenges in epilepsy care, particularly in drug-resistant forms. Digital health tools may improve self-management by integrating seizure tracking, adherence support, and patient–clinician communication. This narrative review summarizes current mobile applications for seizure monitoring and adherence and outlines opportunities and gaps in clinical translation. **Methods:** A narrative synthesis (PubMed, Scopus, Google Scholar; 2019–2025; English) summarized functionality, usability, clinical validation, and limitations of epilepsy-focused mobile/wearable applications; no systematic methods or meta-analysis were applied. Results: Existing tools cluster into seizure diary apps, smartwatch-based monitoring systems, and adherence-focused applications. While they improve documentation and treatment regularity, most lack adaptive personalization, language localization and therapeutically active components. Comprehensive platforms combining tracking, adherence analytics and telehealth remain unevenly validated. Validated wearable detectors for generalized tonic–clonic seizures typically report sensitivity in the 80–95% range in real-world or simulated-real-world studies, alongside variable specificity and false-alarm rates, underscoring the need for individualized deployment and calibration. **Conclusions:** Mobile and wearable applications are promising adjuncts to routine epilepsy care. The field is gradually shifting from passive monitoring toward integrated, user-centered platforms that blend monitoring, predictive analytics and neuromodulation. This review also briefly outlines a conceptual example of an integrated mobile platform that combines seizure documentation, adherence support and patient-initiated rhythmic cueing; this example is presented at a purely exploratory level and requires further clinical validation.

## 1. Introduction

Epilepsy is one of the most common and stigmatizing chronic neurological disorders, affecting more than 50 million people worldwide according to the World Health Organization (WHO, 2024) [1]. Despite the continuous emergence of new anticonvulsants and advances in epilepsy surgery, approximately 30–40% of patients continue to experience seizures, leading to the development of drug-resistant epilepsy (DRE) [2]. In this population, long-term treatment goals extend beyond seizure control and include improving medication adherence, functional independence, and overall quality of life.

Medication non-adherence is recognized as one of the leading causes of poor seizure control, recurrent hospitalizations, and sudden unexpected death in epilepsy (SUDEP) [3]. According to various studies, between 30% and 50% of individuals with epilepsy do not fully adhere to their prescribed antiepileptic drug (ASM) regimens [4]. The causes of poor adherence are multifactorial, including cognitive difficulties, polytherapy, adverse effects, complex dosing schedules, and psychosocial stressors. Thus, improving adherence has become not only a medical but also a behavioral and digital health challenge.

Another important issue in epilepsy management is the accurate documentation of seizures. In routine clinical practice, neurologists and epileptologists rely heavily on patient self-reports or caregiver observations, which are often incomplete and prone to recall bias [5]. Nocturnal or unrecognized seizures may remain unreported, leading to an underestimation of seizure frequency and suboptimal therapy adjustments. In this context, mobile and wearable technologies have emerged as valuable tools for real-time seizure recording and data exchange between patients and physicians.

Over the past decade, the rapid development of digital health technologies has significantly transformed chronic disease management. Mobile health (mHealth) applications are increasingly being integrated into epilepsy care, providing opportunities for self-monitoring, automated medication reminders, data visualization, and interactive feedback [6,7,8]. However, despite these advances, existing digital solutions demonstrate substantial variability in clinical validation, usability, and accessibility. Most applications lack language localization, adaptive personalization, and mechanisms aimed at preventing seizure generalization.

Given these limitations, it is both timely and necessary to analyze the current state of digital tools designed for epilepsy management, emphasizing their functional capabilities and existing gaps. The present narrative review aims to summarize available mobile applications for seizure monitoring and patient self-management, evaluate their role in enhancing medication adherence and quality of life, and to briefly illustrate these concepts using a prototype mobile platform developed by our group; this example is presented at a purely conceptual level.

This article is a narrative, non-systematic review. Examples were selected to illustrate major functional categories of epilepsy applications based on recency, clinical relevance and availability of peer-reviewed or manufacturer documentation; the list is illustrative, not exhaustive.

### Literature Search Strategy (Narrative, Non-Systematic)

This review used a narrative, non-systematic approach. We searched PubMed and Scopus, supplemented by targeted Google Scholar queries, for English-language publications from January 2019 to November 2025 using combinations of the terms: “epilepsy”, “mobile health”, “mHealth”, “seizure diary”, “wearable”, “seizure detection”, “adherence”, “self-management”, “telehealth”, “AI”, and “seizure forecasting”. We prioritized peer-reviewed clinical studies, methodological overviews, and implementation reports; manufacturer white papers were considered only to clarify device functions and were not used as primary evidence. Inclusion criteria were relevance to digital tools for seizure monitoring, adherence support, or patient–clinician communication; exclusion criteria were non-neurologic applications, narrative pieces without technical/clinical content, and duplicate reports. Study selection and data extraction were performed by the author; findings were synthesized qualitatively and organized by functional categories (diaries, wearables, adherence, integrated platforms). No formal risk-of-bias assessment or meta-analysis was performed.

Because of the narrative design, the search was not intended to be exhaustive, and no formal PRISMA procedures, risk-of-bias assessment or quantitative synthesis were performed. The examples included are illustrative and focus on clinically relevant and widely discussed tools.

During manuscript preparation, an AI-based language model (ChatGPT 4.0, OpenAI, San Francisco, CA, USA) was used solely to assist with grammar, style, and formatting. The model did not generate or select scientific content, did not influence the interpretation of the literature, and did not affect the conclusions of this narrative review. All sources, analyses, and interpretations were performed and verified by the author.

## 2. Digital Transformation in Epilepsy Management

In recent years, digital transformation has become a defining trend in modern neurology, reshaping the ways clinicians and patients interact, monitor, and manage chronic neurological diseases. The growing adoption of mobile health (mHealth) and digital health solutions has expanded opportunities for personalized monitoring, patient education, and therapeutic adherence across various neurological domains, including epilepsy, Parkinson’s disease, and multiple sclerosis [9,10].

Within the field of epilepsy, mHealth technologies aim to complement standard therapy by improving seizure documentation, facilitating real-time monitoring, and supporting continuous communication between patients and healthcare providers. The key functional directions of digital solutions can be summarized as follows:

(1) Seizure tracking—digital seizure diaries and automated event logging improve data accuracy and allow patients to visualize seizure dynamics over time [5];
(2) Medication reminders—mobile-based alerts promote adherence to antiepileptic drug regimens, reducing missed doses and improving seizure control [4];
(3) Wearable seizure detection—integrating accelerometers, electrodermal sensors, and EEG headbands enables early identification of tonic–clonic seizures and nighttime events [11,12];
(4) Patient–clinician communication—secure data sharing platforms enhance treatment individualization and facilitate remote follow-up [6].

A major paradigm shift in recent years involves the transition from passive monitoring to active seizure management. Earlier applications primarily served as digital logs for seizure frequency and medication intake, whereas contemporary solutions increasingly incorporate predictive algorithms, biofeedback, and multimodal sensor integration to anticipate and prevent seizure generalization [13]. This evolution mirrors broader digital medicine trends, in which continuous data collection and machine learning are used to detect subtle preictal changes in physiology, offering opportunities for early intervention [14].

Ultimately, digital transformation in epilepsy management represents a move toward a proactive, patient-centered model of care, in which real-time analytics, adherence support, and behavioral guidance converge to empower patients and reduce disease burden.

## 3. Overview of Existing Epilepsy Applications

A growing number of mobile and wearable applications have been developed to assist patients with epilepsy in monitoring seizures, tracking medication intake, and communicating with healthcare providers. Although their overall goal is to improve disease management and patient autonomy, these tools vary considerably in functionality, accessibility, and clinical validation. Table 1 provides an overview of the most widely used and emerging epilepsy-related applications, grouped by their primary focus.

While Table 1 outlines the main functional categories of digital tools for epilepsy management, these applications differ substantially in validation depth and regulatory maturity.

Table 2 provides an overview of key validation and performance data where available.

### 3.1. Clarifying Evidence Sources and Validation Levels

When describing the functionalities of digital tools, including EpiTapp^®^ and other applications, it is important to note that certain features are documented primarily through developer-reported descriptions and publicly available technical specifications. These descriptions reflect intended functions rather than independently verified clinical performance.

Where available, we explicitly refer to peer-reviewed validation studies, usability assessments, or real-world evaluations. However, for several applications, published evidence remains limited, and functional claims are based on manufacturer documentation rather than empirical testing. Distinguishing between function-level descriptions and evidence-based validation helps avoid overinterpretation and ensures appropriate contextualization for clinicians and researchers.

### 3.2. Narrative Analysis

Seizure diary applications such as Seizure Tracker, EpiDiary, and My Seizure Diary remain the most widely used digital tools among people with epilepsy. These platforms allow users to manually document seizures, triggers, and medication use, and to share reports with physicians. While they improve recall accuracy compared with paper diaries, their reliance on self-reporting limits data completeness and objectivity [15,16].

Smartwatch-based systems, including EpiWatch (Apple Inc., Cupertino, CA, USA) and Empatica Embrace2 (Empatica, Boston, USA/Milan, Italy), introduced sensor-driven monitoring, leveraging accelerometers, gyroscopes, and electrodermal activity to detect convulsive seizures. Clinical studies have confirmed their feasibility and moderate accuracy, though cost and limited access remain barriers to widespread use [11,17]. Epihunter offers a complementary approach by detecting absence seizures via EEG-based attention monitoring, enhancing early detection in classroom or workplace settings [18].

Medication adherence apps such as Medisafe and MyTherapy are general digital therapeutics supporting chronic disease management through medication reminders, adherence analytics, and patient feedback [19]. Although not epilepsy-specific, these tools have suggested positive effects on treatment regularity and patient satisfaction [4].

Comprehensive epilepsy self-management platforms, including Helpilepsy and Seer Medical, represent the next generation of integrated systems. They combine seizure tracking, adherence support, and remote physician connectivity. Helpilepsy offers an intuitive patient dashboard and clinician portal, whereas Seer Medical integrates long-term video-EEG data collection and AI-based seizure classification [20,21]. The NeuroVista platform, still experimental, explores implantable EEG sensors for real-time seizure forecasting [25,26,27].

Despite these advances, most applications lack adaptive personalization, cultural and linguistic localization, or built-in algorithms for active seizure prevention [28,29,30,31]. This underscores the need for next-generation systems that integrate both monitoring and assistive interventions, bridging the gap between observation and action.

As one illustration of an integrated approach, a prototype mobile platform (EpiTapp^®^) has been developed to combine seizure documentation, medication reminders and a module for patient-initiated 1 Hz vibrotactile cueing during early focal seizure sensations. In a small exploratory study, delivery of such rhythmic cues via a smartphone interface was associated with short-term modulation of interhemispheric EEG coherence without adverse events [32]. These data are preliminary, involve a limited sample, and do not establish clinical efficacy; the example is included solely to illustrate how monitoring, adherence support and neuromodulation could theoretically be bundled within a single tool alongside other digital solutions. A schematic example of a possible integrated architecture is provided in Supplementary Figure S1.

Similar conceptual integrations are being explored in other digital neurology domains, and future work will need to determine whether such combined platforms provide measurable benefits beyond standard seizure diaries and adherence apps.

### 3.3. Neurophysiological Basis of Rhythmic Sensory Cueing

Slow rhythmic sensory stimulation (~1 Hz) is hypothesized to transiently engage large-scale timing and prediction networks that rely on low-frequency oscillatory coordination. Studies in cognitive neuroscience demonstrate that 1 Hz external pacing can entrain delta–theta band activity, enhance temporal prediction, and stabilize attentional control through resonance mechanisms in prefrontal and fronto-insular circuits. These regions are critically involved in processing interoceptive signals, sustaining cognitive control, and regulating the early spread of focal epileptic activity. While speculative in the context of epilepsy, such entrainment-based mechanisms provide a plausible framework for understanding how slow, predictable rhythmic cues might momentarily modulate neural timing and attentional focus during early seizure sensations. This rationale does not imply therapeutic efficacy but serves only as a mechanistic hypothesis consistent with existing entrainment research. At present, the clinical relevance of such mechanisms for routine epilepsy management remains speculative.

Several other mobile and wearable systems—such as Seizure Tracker and My Seizure Diary for seizure logging, Medisafe and MyTherapy for adherence support, and wearable detectors like Empatica Embrace2—also contribute to the ecosystem of epilepsy-focused digital tools. EpiTapp^®^ belongs to this broader group and differs primarily in its conceptual incorporation of slow rhythmic cueing as an assistive component, which has yet to be clinically validated. This contextualization is intended to minimize any appearance of promotional emphasis and to position EpiTapp^®^ alongside comparable solutions rather than above them.

## 4. Limitations and Gaps in Current Digital Solutions

Despite substantial progress in the development of mobile and wearable technologies for epilepsy management, current digital solutions face several conceptual and practical limitations that restrict their clinical applicability and long-term sustainability. These shortcomings can be broadly categorized into five domains: technological constraints, limited personalization, poor adherence integration, insufficient clinical validation, and lack of active therapeutic support.

### 4.1. Evidence Strength and Methodological Heterogeneity Across Digital Tools

The evidence base supporting digital tools for epilepsy is heterogeneous, with substantial variability in study design, sample size, validation endpoints, and real-world generalizability. Wearable seizure detection systems—such as Empatica Embrace2—have undergone multi-center real-world validation and report sensitivity estimates of 80–95% for generalized tonic–clonic seizures, but these findings are highly dependent on population characteristics, device calibration, and nocturnal monitoring conditions. However, validation cohorts frequently over-represent adults with generalized tonic–clonic seizures treated at tertiary epilepsy centers, while pediatric populations, focal non-motor seizures and individuals from low-resource settings are under-represented. This sampling bias limits the generalizability of reported performance metrics. In contrast, most seizure diary applications and adherence platforms rely primarily on self-reported outcomes without controlled validation studies, limiting the interpretability of performance metrics. Research-use smartwatch systems (e.g., EpiWatch) frequently depend on participant-driven logging and therefore introduce reporting bias.

Furthermore, many studies are conducted in simulated or semi-controlled environments rather than fully naturalistic contexts, which may overestimate device performance. Sample sizes vary widely—from small feasibility cohorts (*n* < 20) to larger observational datasets—which contributes to inconsistent reliability across studies. Randomized controlled trials remain rare, and standardized validation frameworks for digital seizure detection, adherence assessment, and multimodal integration are lacking.

Given these discrepancies, comparative interpretation must be made cautiously. Future studies require harmonized protocols, independent validation, and real-world, diverse cohorts to ensure reproducibility and equitable applicability.

First, most existing applications are designed for data collection rather than therapeutic engagement. They focus primarily on passive monitoring—recording seizures, medication schedules, and symptoms—without providing tools that can directly influence seizure dynamics or behavioral outcomes. While seizure diaries enhance communication between patients and physicians, they do not offer mechanisms to prevent or mitigate seizure generalization in real time [6].

Second, the majority of applications lack individualized adaptation to patient-specific factors. Variations in seizure type, comorbidities, lifestyle, and cognitive function are rarely considered in interface design or notification logic. Studies show that generic reminder systems, although effective for medication timing, often fail to sustain engagement beyond the initial months of use [23,24,25]. This highlights the importance of integrating behavioral reinforcement and personalization algorithms into digital health tools.

Across multiple mHealth domains, a substantial proportion of users discontinue regular app use within the first months after download, leading to marked “app attrition”. This pattern likely reduces the real-world effectiveness of adherence and self-management tools, even when short-term usability ratings are high.

Third, there remains an absence of integrated adherence analytics. Medication adherence is often treated as a separate task from seizure tracking, even though these parameters are interdependent in determining seizure control. Multimodal integration—combining adherence data, seizure logs, and physiological indicators—could provide a more accurate picture of treatment effectiveness but is rarely implemented in commercial apps [25].

Fourth, clinical validation and regulatory compliance remain uneven. Many applications in use today have not undergone formal clinical testing or peer-reviewed evaluation. Only a limited subset, such as Empatica Embrace2 or Seer Medical, has suggested diagnostic reliability under controlled conditions [11,21]. Furthermore, issues related to data privacy, interoperability, and ethical data sharing continue to pose significant challenges [26].

Finally, few digital tools incorporate active therapeutic components capable of supporting neurophysiological modulation or behavioral stabilization. Recent trends in digital neurology emphasize the shift toward closed-loop systems and responsive neurostimulation paradigms, which use real-time data to guide intervention [31]. However, the integration of such approaches into patient-operated mobile platforms remains in its infancy.

These limitations underscore the need for next-generation digital health applications that move beyond passive tracking toward interactive, assistive, and adaptive solutions. Prototype hybrid systems that combine seizure monitoring, adherence support and neuromodulatory components—such as those explored in our previous work [27]—illustrate a conceptual pathway toward bridging the gap between digital observation and active clinical assistance, but their clinical utility remains to be demonstrated.

A proportion of the functional characteristics of epilepsy-related mobile applications are derived from publicly available developer documentation. While such information is useful for understanding intended use, empirical validation varies widely across tools. Therefore, functional descriptions are presented separately from evidence-based findings to avoid conflating technical capability with proven clinical performance.

### 4.2. Global Accessibility, Language Localization, and Health Equity

Global accessibility remains a critical and often overlooked limitation of current epilepsy-related digital tools. Most commercially available mobile applications are developed primarily for English-speaking users and offer limited or no localization into other languages. This lack of linguistic diversity presents substantial barriers for individuals in low-resource, rural, or non-English-speaking regions, where epilepsy prevalence is high and access to specialized care is limited.

Beyond language, cultural adaptation is rarely addressed: user interfaces, educational content, and adherence reminders often reflect Western healthcare contexts and may not align with regional health practices or literacy levels. Studies in digital health indicate that inadequate localization directly reduces user engagement, adherence to digital interventions, and sustained app utilization, ultimately limiting potential clinical impact.

Furthermore, the unequal distribution of smartphones, internet access, and wearable sensors reinforces existing health inequities. Resource-limited settings may lack affordable access to validated seizure detection devices, cloud-based analytical services, or subscription-based applications. Thus, without deliberate efforts to improve localization, affordability, and offline-capable design, the benefits of digital epilepsy management tools may remain restricted to a narrow patient population.

Addressing these barriers requires coordinated development of multilingual interfaces, culturally adapted education modules, low-bandwidth operation, and pricing models that support equitable access across diverse global populations.

These challenges are particularly pronounced in low- and middle-income countries, where smartphone penetration, broadband coverage and access to wearable sensors may be limited, and where out-of-pocket costs for commercial devices can be prohibitive. Without deliberate design for low-resource environments, digital epilepsy tools risk widening, rather than closing, existing gaps in care.

#### Regulatory, Privacy, and Interoperability Considerations

In routine deployment, epilepsy apps must comply with jurisdiction-specific privacy frameworks (e.g., GDPR in the EU and HIPAA in the US), including lawful bases for processing, data minimization, transparent consent, secure at-rest/in-transit encryption, and clear policies on cross-border transfers and third-party processors. Interoperability with EHRs (HL7/FHIR) remains uneven and can limit clinical integration. For AI-enabled features, model transparency, auditability, and post-deployment monitoring for drift and bias are necessary to maintain safety and trust. These requirements add operational complexity but are essential for scalable and equitable digital neurology.

## 5. Future Directions and Perspectives

The rapid advancement of digital health and computational neuroscience is reshaping the landscape of epilepsy management. Future directions point toward the convergence of multimodal data integration, artificial intelligence (AI)-based analytics, and adaptive neurostimulation—moving from descriptive monitoring toward proactive, closed-loop intervention systems.

### 5.1. Integration of Multimodal Data Sources

Emerging digital platforms are increasingly combining heterogeneous data streams—self-reported seizures, medication adherence, wearable biosignals, and environmental factors—to generate individualized seizure risk profiles [27]. Such integrative frameworks enhance diagnostic precision and allow continuous patient monitoring beyond clinical settings. Cloud-based analytics enable dynamic feedback loops between patients, caregivers, and physicians, providing a foundation for personalized treatment adjustments [28].

### 5.2. AI and Predictive Modeling in Seizure Forecasting

Recent studies have suggested that AI algorithms trained on EEG, actigraphy, and physiological parameters can predict seizure likelihood hours in advance with clinically meaningful accuracy [29]. The transition from retrospective seizure detection to real-time forecasting represents a paradigm shift, enabling early-warning notifications and lifestyle adaptation. However, challenges related to data quality, generalizability, and algorithm transparency persist and require standardization across platforms [30].

### 5.3. Closed-Loop and Responsive Neuromodulation

Closed-loop neuromodulatory systems—such as adaptive vagus nerve stimulation (aVNS) and responsive neurostimulation (RNS)—have shown promising results in reducing seizure frequency through real-time detection and targeted intervention [31]. Translating these principles into mobile or wearable applications remains an emerging frontier. The concept of exogenous rhythmic stimulation (ERS), as implemented in patient-initiated mobile systems, provides an accessible pathway for user-controlled neuromodulation without implanted devices, aligning with this paradigm [12,27].

### 5.4. Behavioral and Cognitive Digital Therapeutics

Beyond seizure control, the next generation of digital interventions aims to address cognitive and emotional dimensions of epilepsy. Integrating cognitive-behavioral therapy (CBT), mindfulness, and anxiety management tools into epilepsy apps has been shown to improve adherence and perceived quality of life [33,34]. This approach emphasizes the holistic nature of digital neurology—supporting both neurological stability and psychological resilience.

### 5.5. Interoperability, Ethics, and Equity

As digital ecosystems expand, issues of interoperability, data privacy, and equitable access gain increasing importance. The development of transparent AI frameworks, open data repositories, and regulatory harmonization will be essential to ensure reproducibility and patient trust [35]. Furthermore, global initiatives are needed to adapt epilepsy apps for low-resource settings and diverse linguistic environments.

In summary, the future of epilepsy management is likely to involve integrated, adaptive and participatory digital systems that unify monitoring, prediction and intervention within a single clinical continuum. Hybrid mobile applications that combine seizure tracking, adherence support and real-time neuromodulation represent a possible step toward this vision; at present, such approaches remain conceptual and will require rigorous, controlled evaluation before routine clinical use can be recommended.

## 6. Conclusions

Digital health technologies are reshaping the management of epilepsy, offering tools that extend beyond traditional pharmacological treatment. Current mobile and wearable applications facilitate seizure tracking and adherence monitoring but remain limited by insufficient personalization, clinical validation, and therapeutic interactivity. The evolution toward integrated, adaptive, and user-centered platforms—combining monitoring, predictive analytics, and neuromodulation—represents an emerging direction in digital epileptology. Such approaches, including prototype hybrid systems that combine monitoring, adherence support and neuromodulation, may enhance patient autonomy, treatment adherence and quality of life in the future, provided that ongoing research confirms their safety, feasibility and cost-effectiveness.

## Figures and Tables

**Table 1:** Functional overview of current epilepsy management applications. Brand names are illustrative and do not imply endorsement.

Category	Example Applications	Primary Functions	Limitations	References
Seizure Diary Apps	Seizure Tracker (Seattle, WA, USA), Epilepsy Journal (USA), EpiDiary (Lundbeck) (Copenhagen, Denmark), My Seizure Diary (Epilepsy Foundation) (Landover, MD, USA)	Manual logging of seizures (type, duration, triggers), exportable reports, graphical visualization of seizure trends	Lack of automated detection; limited analytics; mostly English interface	[5,15,16]
Smartwatch-based Monitoring	EpiWatch (Apple Inc.) (Cupertino, CA, USA), Empatica Embrace2 (Boston, USA/Milan, Italy), Epihunter (Leuven, Belgium)	Automatic seizure detection using accelerometry, electrodermal activity, or EEG; real-time alerts to caregivers	High device cost; limited OS compatibility; variable sensitivity/specificity	[17,18]
Adherence-focused Apps	Medisafe (Boston, MA, USA), MyTherapy (Munich, Germany)	Personalized medication schedules, push notifications, intake confirmation, adherence reports	Not epilepsy-specific; no integration with seizure tracking	[4,19]
Comprehensive Apps for Epilepsy Self-Management	Helpilepsy (Brussels, Belgium), Seer Medical (Melbourne, Australia), NeuroVista (experimental) (Seattle, WA, USA)	Combines seizure diary, medication tracking, telehealth communication, and clinician dashboard	Limited localization and personalization; some require subscription or clinical enrollment	[20,21,22,23,24,25]

**Table 2:** Validation status, regulatory approval, performance characteristics, and key limitations of selected epilepsy-related digital tools.

Application/ Platform	Category	Regulatory Status (FDA/CE)	Validation Study (Ref.)	Reported Sensitivity/ Specificity	Target Seizure Type	Limitations
Empatica Embrace2 (iOS/Android)	Wearable detector	FDA 510 (k)-cleared wearable for convulsive seizure alerting; validated in multi-site real-world studies.	Aziz et al., J. Med. Internet Res. 2025 [11]	Sens. ≈ 92%, Spec. ≈ 85%	GTC	Validated mainly for generalized tonic–clonic seizures; variable specificity and false-alarm rates; requires subscription and is less informative for non-motor seizures.
EpiWatch (Johns Hopkins/Apple ResearchKit) iOS (Apple Watch)	Smartwatch research app	Research use only (not FDA—cleared)	Aziz et al., Wearable Artificial Intelligence for Epilepsy: Scoping Review. J. Med. Internet Res. 2025 [11]	Feasibility studies, self-reported seizure data	GTC/focal	Feasibility-only evidence; relies on self-report; not an FDA-cleared detector.
Epihunter (Android/Web)	EEG headset + app	Feasibility for absence seizures; not FDA-cleared; consumer CE-marked device	Epihunter NV, company documentation [8]	Feasibility for absence seizures	Absence	Limited validation; feasibility data only; requires external EEG headset; applicability restricted to absence seizures.
Helpilepsy (iOS/Android + portal)	Comprehensive self-management	Clinical use reports only, not classified as a medical device	J. Med. Internet Res. 2020 [23]	Usability > 80% user satisfaction	All	No formal clinical validation; relies on manual entries; functionality varies by subscription level.
Seer Medical (Web portal/cloud)	Long-term ambulatory EEG	Regulated diagnostic platform	Biondi et al., EEG@HOME protocol; Tang et al., wearable seizure detection review [21,26,27,28,29]	Diagnostic accuracy > 95% for EEG capture	All	Requires clinical referral; high cost; usability not generalizable to standard consumer devices.
Medisafe (iOS/Android)	Adherence support app	Not medical device	J. Manag. Care Spec. Pharm. 2020 [19]	Improved adherence ~15–20% in meta-analysis	All	Not epilepsy-specific; no seizure tracking; evidence mainly from chronic disease populations.
My Therapy (iOS/Android)	Adherence support app	Not medical device	Epilepsia 2023 [28]	Improved medication regularity	All	Not epilepsy-specific; limited integration with seizure data; effectiveness depends on user engagement.
EpiDiary (Lundbeck) (iOS/Android)	Seizure diary	Pharma-sponsored app	Sci. Rep. 2024/Neurology 2018 [5,16]	Improves reporting accuracy vs. paper	All	Manual reporting only; lacks automated detection; potential for incomplete data.
My Seizure Diary (Web/mobile)	Seizure diary (Foundation)	Non-regulated	My Seizure Diary report 2024 [15]	Improved patient engagement > 70%	All	Self-report only; no validation studies confirming accuracy; limited personalization.
NeuroVista (Implant + external)	Implanted forecasting system	Former clinical trial (Lancet Neurol 2013)	Cook et al., Lancet Neurol. 2013 [22]	Forecast accuracy up to 70%	Focal/DRE	Implantable research system; no commercial availability; evidence limited to small trials.

All performance values are approximate and derived from published or reported data; provided for narrative illustration only.

## Data Availability

No new data were created or analyzed in this study; data sharing does not apply to this article.

## Supplementary Materials

The following supporting information can be downloaded at: https://www.mdpi.com/article/10.3390/jcm14248701/s1, Figure S1: Conceptual structure of an integrated epilepsy self-management app, illustrating the combination of seizure documentation, adherence tracking and a user-triggered neuromodulation module.
