# Supplementary material for: Digital Tools for Seizure Monitoring and Self-Management in Epilepsy: A Narrative Review
Source: J Clin Med. 2025 Dec 9;14(24):8701. doi: 10.3390/jcm14248701 (PMC12733385; doi:10.3390/jcm14248701)
Supplement: Supplementary file 1 [file jcm-14-08701-s001.zip › jcm-4010733-supplementary.pdf]

Supplementary

Figure S1

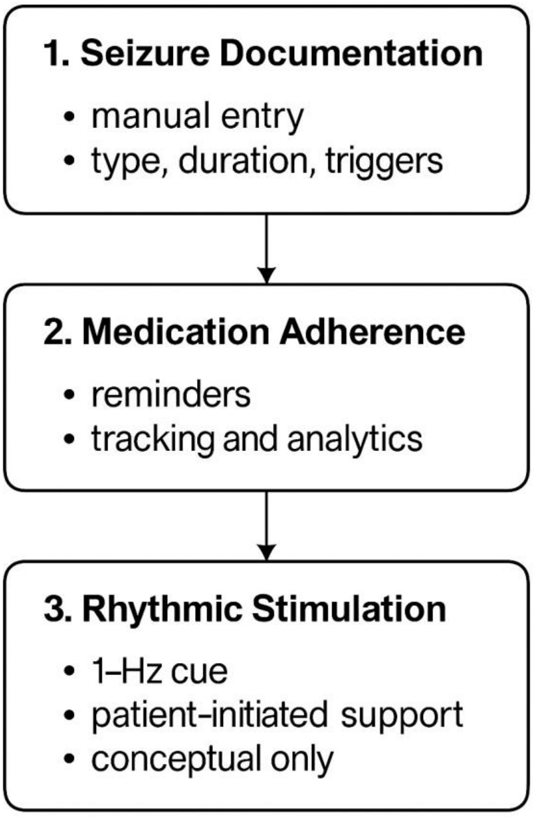

Supplementary Figure S1. Conceptual structure of an integrated epilepsy self-management app, illustrating the combination of seizure documentation, adherence tracking and a user-triggered neuromodulation module. The schematic is generic and not intended as promotional material.
